# Diagnostic Accuracy of Deep Learning and Radiomics in Lung Cancer Staging: A Systematic Review and Meta-Analysis

**DOI:** 10.3389/fpubh.2022.938113

**Published:** 2022-07-18

**Authors:** Xiushan Zheng, Bo He, Yunhai Hu, Min Ren, Zhiyuan Chen, Zhiguang Zhang, Jun Ma, Lanwei Ouyang, Hongmei Chu, Huan Gao, Wenjing He, Tianhu Liu, Gang Li

**Affiliations:** ^1^Department of Thoracic Surgery, The 3rd Affiliated Hospital of Chengdu Medical College, Pidu District People's Hospital, Chengdu, China; ^2^School of Electronic Engineering, Chengdu University of Technology, Chengdu, China; ^3^Department of Cardiology, The 3rd Affiliated Hospital of Chengdu Medical College, Pidu District People's Hospital, Chengdu, China

**Keywords:** lung cancer, deep learning, radiomics, diagnostic accuracy, lymph node metastasis, meta-analysis

## Abstract

**Background:**

Artificial intelligence has far surpassed previous related technologies in image recognition and is increasingly used in medical image analysis. We aimed to explore the diagnostic accuracy of the models based on deep learning or radiomics for lung cancer staging.

**Methods:**

Studies were systematically reviewed using literature searches from PubMed, EMBASE, Web of Science, and Wanfang Database, according to PRISMA guidelines. Studies about the diagnostic accuracy of radiomics and deep learning, including the identifications of lung cancer, tumor types, malignant lung nodules and lymph node metastase, were included. After identifying the articles, the methodological quality was assessed using the QUADAS-2 checklist. We extracted the characteristic of each study; the sensitivity, specificity, and AUROC for lung cancer diagnosis were summarized for subgroup analysis.

**Results:**

The systematic review identified 19 eligible studies, of which 14 used radiomics models and 5 used deep learning models. The pooled AUROC of 7 studies to determine whether patients had lung cancer was 0.83 (95% CI 0.78–0.88). The pooled AUROC of 9 studies to determine whether patients had NSCLC was 0.78 (95% CI 0.73–0.83). The pooled AUROC of the 6 studies that determined patients had malignant lung nodules was 0.79 (95% CI 0.77–0.82). The pooled AUROC of the other 6 studies that determined whether patients had lymph node metastases was 0.74 (95% CI 0.66–0.82).

**Conclusion:**

The models based on deep learning or radiomics have the potential to improve diagnostic accuracy for lung cancer staging.

**Systematic Review Registration:**

https://inplasy.com/inplasy-2022-3-0167/, identifier: INPLASY202230167.

## Introduction

Lung cancer is one of the most common malignancies globally and the leading cause of cancer-related death in the world. Its morbidity and cancer-related mortality rank first among malignant tumors. There are ~2.2 million new cases and about 1.5 million deaths worldwide (1).

Radiomics and deep learning, as an innovative means to characterize lung lesions, can be applied to generate descriptive data, build predictive model, and correlate quantitative image features with phenotypes or gene-protein signatures, thus aiding in cancer detection, diagnosis, staging, treatment response prediction, and prognosis assessment and playing an increasingly important role in clinical decision-making, especially the management of malignant tumors (2).

Lung cancer staging is usually done by radiologists evaluating CT images of patients with lung cancer. The accuracy of diagnosis is affected by various factors, such as device performance, standardized imaging protocols, the experience of the reporting radiologist, and patient-specific factors. While radiomics involves using advanced computational algorithms to extract large numbers of researcher-defined features from images for defining related lung lesions, studies suggesting that deep learning algorithms can identify a more nuanced approach that eschews traditional radiology and statistical methods for cancer staging were extensively reported (3–6). Deep learning, as a new research direction in the field of machine learning (ML), is applied to learn the inherent laws and representation levels of sample data for feature recognition and model building (7). In the last decade, radiomics models and deep learning have made meaningful contributions to medical imaging diagnosis and related individual medicine (8).

This study aimed to perform a systematic review and meta-analysis of published data on lung cancer diagnosis and the diagnostic accuracy of deep learning algorithms and radiomics models for lung cancer staging.

## Methods

### Search Strategy

This study followed the Preferred Reporting Item of the Guidelines for Systematic Reviews and Meta-Analysis (PRISMA), and selection criteria, data extraction, and data analysis were determined before study initiation. Any eligible studies in the PubMed, EMBASE, Web of Science, and Wanfang Database will be searched by Cancer, Radiomics, Deep Learning, Lung Cancer, and more. The search method is shown in Table 1. Search terms such as “radiomics,” “deep learning,” “lymph node metastasis,” “non-small cell lung cancer,” “malignant lung nodules,” and “diagnostic accuracy.” Use the Boolean operator AND to combine the results of different queries. We also manually searched the reference lists of included studies to identify any relevant articles. Both English and Chinese articles are considered eligible.

**Table 1:** Search strategy.

**Sources**	**Search in**	**MeSH terms**	**Limits**	**Search results**
Web of science	Search manager	(“deep learning” OR “convolutional neural network” OR “machine learning” OR “radiomics” OR “radiomic”) AND (“CT” OR “MRI”) AND (“Lymph node” OR “lymph node metastasis” OR “Benign and malignant pulmonary nodules”)AND (“lung cancer” OR “non-small cell lung cancer” OR “NSCLC”)	None	11
PubMed, (MEDLINE)	N/A	(“deep learning” OR “convolutional neural network” OR “machine learning” OR “radiomics” OR “radiomic”) AND (“CT” OR “MRI”) AND (“Lymph node” OR “lymph node metastasis” OR “benign and malignant pulmonary nodules”) AND (“lung cancer” OR “non-small cell lung cancer” OR “NSCLC”)	None	30
EMBASE	Quick search	(‘deep learning'/exp OR “deep learning” OR “machine learning”/exp OR “machine learning” OR “radiomics”/exp OR “radiomics” OR “radiomic”) AND (“ct”/exp OR “ct” OR “mri”/exp OR “mri”) AND (“lymph node”/exp OR “lymph node” OR “lymph node metastasis”/exp OR “lymph node metastasis” OR “benign and malignant pulmonary nodules”) AND (“lung cancer”/exp OR “non-small cell lung cancer” OR “NSCLC”)	None	56
Wanfang database	N/A	(“deep learning” OR “machine learning” OR “radiomics” OR “radiomic”) AND (“CT” OR “MRI”) AND (“Lymph node” OR “lymph node metastasis”) AND (“lung cancer” OR “NSCLC”)	None	5

### Study Selection

We selected publications for review if they met several of the following inclusion criteria: (1) patients with pathologically diagnosed lung cancer were included in the study; (2) radiomics or deep learning algorithms applied to lung cancer staging were evaluated. Exclusion criteria included: (1) informal publication types (e.g., reviews, letters to the editor, editorials, conference abstracts); (2) only focus on research on image segmentation or image feature extraction methods; (3) animal studies. After the removal of duplicates, titles and abstracts were identified by two independent reviewers using the Covidence systematic review software. Any disagreements will be resolved by consensus by arbitration by a third author.

### Data Extraction

We reviewed data from selected primary studies using standardized forms, and two reviewers independently extracted data from each eligible study. Data extraction for each study included first author, country, year of publication, type of AI model, number of patients, patient characteristics (mean/median age, gender), type of malignancy, benign and malignant pulmonary nodules, lymph node metastasis. In addition, we extracted the area under the receiver operating characteristic curve (AUROC), along with sensitivity, specificity, accuracy, etc., for data processing and forest map production. The primary endpoint of this systematic review was AUROC.

### Quality Assessment

Two independent reviewers will initially assess the risk of bias. A third reviewer will then review each study using the Quality Assessment of Studies for Diagnostic Accuracy (QUADAS-2) guidelines. The QUADAS-2 tool can assign a risk of bias rating of “low,” “high,” or “uncertain” based on the answer to “yes,” “no,” or “uncertain” to the relevant flag questions included in each section. For example, if the answer to all the landmark questions in a range is “yes,” then it can be rated as low risk of bias; if all the informational questions are answered “no,” then the risk of bias is rated as “high” (9). We summarized the risk of bias in individual studies in a narrative summary during the systematic review phase.

### Statistical Analysis

The accuracy measures for this diagnostic meta-analysis included pooled sensitivity, pooled specificity, and their 95% confidence intervals (95% CI). Missing data is calculated using the formula in Table 2. At the same time, AUROC was calculated; an AUROC value close to 1.0 indicates that the test can discriminate almost perfectly, while an AUROC value close to 0.5 means poor discrimination (10, 11). The discordance index (*I*^2^) was used (12). Heterogeneity was assessed as low, medium, and high, with upper limits for *I*^2^ of 25, 50, and 75%, respectively. A forest plot was drawn to show the AUROC estimates relative to the summary pooled estimates for each study. In addition, we will draw a funnel plot to assess publication bias more intuitively. All statistical analyses were performed using STATA V16.0 software.

**Table 2:** Formulas.

**Measure**	**Formula**
Sensitivity	$\frac{TP}{P}\;=\frac{TP}{TP\;+\;FN}$
Specificity	$\frac{TN}{N}=\;\frac{TN}{TN\;+\;FP}$
Accuracy	$\frac{TP\;+\;TN}{P+N}=\;\frac{TP+TN}{TP\;+\;TN\;+\;FP\;+\;FN}$
PPV	$\frac{TP}{TP\;+\;FP}$
NPV	$\frac{TN}{TN\;+\;FN}$
SE	$\frac{\left(UpperLimit-LowerLimit\right)}{3.92}$
95% Confidence interval	*bestestimate* +/− (1.96) * (*SE*)

*P, condition positive; N, condition negative; FN, false negative; FP, false positive; TN, true negative and TP, true positive; PPV, positive predictive value; NPV, negative predictive value; Upper limit, upper limit of confidence interval; Lower limit, lower limit of confidence interval; SE, standard error*.

## Results

### Study Selection

**Figure 1:**
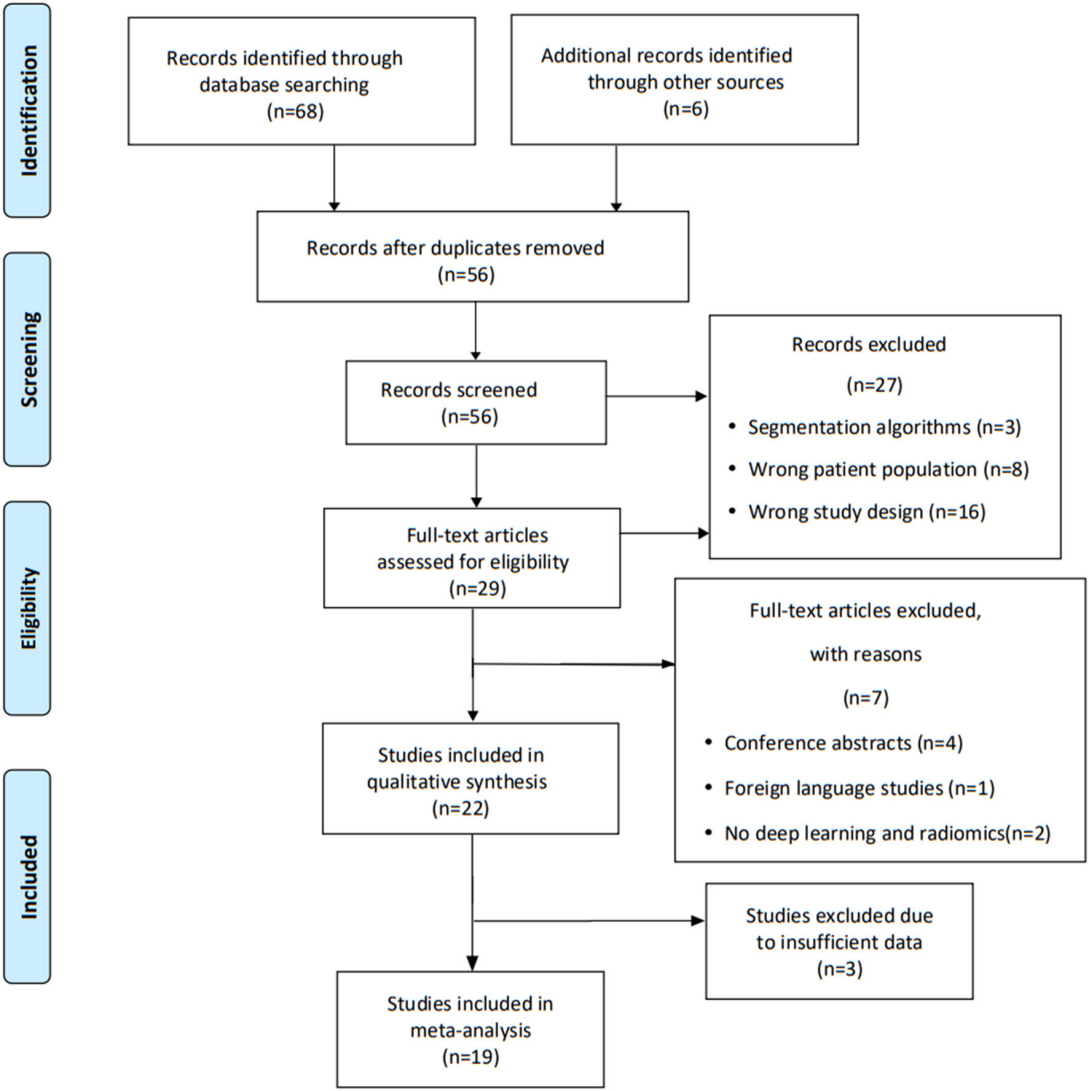
PRISMA flow chart outlining the selection of studies for review.

Our search identified 74 studies, with 56 screened after removing duplicates. Of these, 27 did not meet the inclusion criteria based on title and abstract. The remaining 29 full manuscripts were individually assessed, and, finally, 22 studies were eligible and included in our systematic review. Of these, 19 papers were available for meta-analysis, and five articles were excluded because of their insufficient data information. We outline the study selection process for review using the PRISMA flowchart (Figure 1).

### Study Characteristics

**Figure 2:**
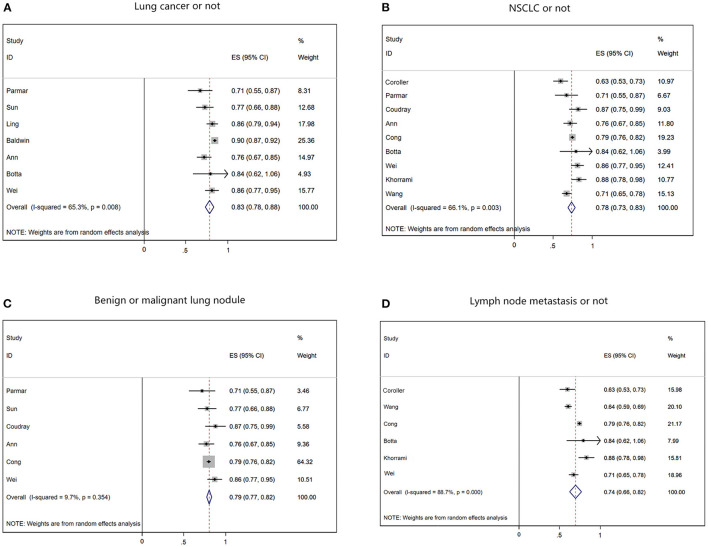
Summary of forest plots for different classifications. **(A)** The forest plot of determine if a patient has lung cancer. **(B)** The forest plot of determining whether the cancer type is NSCLC. **(C)** The forest plot of predicting benign and malignant pulmonary nodules. **(D)** The forest plot of predicting lymph node metastasis in lung cancer.

Of the 19 included studies, 14 had sufficient data for a meta-analysis of AUROC (Figure 2). Regarding study design, 17 studies were retrospective, and two were prospective. Sixteen studies were single-center, and the other three were multicenter. Most of the patients are male, and the median age of 63 years (24–93 years) [Table 3 (13–31)]. The malignancy type in twelve studies was NSCLC, and the malignancy type in the remaining studies was lung cancer. Seven studies used the diagnostic output per patient, and eight studies used the lymph node diagnostic output per node for metastases. While seven studies used post-operative pathology reports as reference standards, 11 used radiology reports.

**Table 3:** Selected characteristics of included studies.

**References**	**Country**	**Year**	**Study design**	**Patients (% female patients)**	**Sample size for diagnostic accuracy**	**Mean or median age (SD; range), years**	**Imaging modality**	**Type of malignancy**	**AI model (Per-patient/per-node diagnostic output)**	**Reference standard**	**Classification criteria**
Coroller et al. (13)	USA	2016	Retrospective single-center	85 (65%)	–	60.3	CT	NSCLC	Radiomics (per-patient)	Radiology	B D
Parmar et al. (14)	USA	2018	Retrospective single-center	1,194	–	68.3 (32–93)	CT	NSCLC	Deep learning (per-patient)	Pathology	A B C
Sun et al. (15)	China	2019	Retrospective single-center	385 (68%)	201	53.1 (±12.2)	CT	Lung Cancer	Radiomics (per-patient)	Radiology	A C
Ling et al. (16)	China	2019	Retrospective multi-center	229 (31.5%)	74	64 (59–81)	CT	Lung Cancer	Radiomics (per-patient)	Radiology	A
Coudray et al. (17)	USA	2018	Retrospective single-center	1,176	459	61 (51.3–72.8)	CT	NSCLC	Deep learning (per-patient)	Radiology	B C
Xu et al. (18)	China	2019	Retrospective single-center	179 (52.8%)	–	63 (32–93)	CT	NSCLC	Deep learning (per-patient)	Pathology	B D
Baldwin et al. (19)	UK	2020	Retrospective single-center	1,337	328	–	CT	Lung Cancer	Deep learning (per-patient)	–	A
Schroers et al. (20)	Germany	2019	Retrospective single-center	82 (38%)	50	61.5 (±5.0)	MRI	Lung Cancer	Radiomics (per-patient)	Pathology	A C
Wang et al. (21)	China	2019	Retrospective single-center	249 (39.8%)	–	61.4 (±8.96)	CT	Lung Cancer	Deep learning (per-patient)	Radiology	D
Leleu et al. (22)	France	2020	Retrospective single-center	215 (39%)	72	58.6 (±10.3)	CT	Lung Cancer	Radiomics (per-patient)	Pathology	A
Ann et al. (23)	USA	2019	Prospective multi-center	262	48	–	CT	NSCLC	Radiomics (per-patient)	Pathology	A B C
Cong et al. (24)	China	2020	Retrospective single-center	411 (50.4%)	141	59.62 (24–84)	CT	NSCLC	Radiomics (per-patient)	Radiology	B C D
Botta et al. (25)	Italy	2020	Retrospective single-center	270 (38%)	–	67.4 (61.0–72.6)	CT	NSCLC	Radiomics (per-patient)	Radiology	A B D
Wei et al. (26)	USA	2020	Retrospective multi-center	146 (39.7%)	–	65.72 (± 12.88)	PET/CT	NSCLC	Radiomics (per-node)	Radiology	A B C
Khorrami et al. (27)	USA	2019	Retrospective single-center	112	–	–	CT	NSCLC	Radiomics (per-patient)	Pathology	B D
Kirienko et al. (28)	Italy	2021	Retrospective single-center	149 (37.6%)	73	70 (41–84)	PET/CT	Lung Cancer	Radiomics (per-node)	Radiology	B C
Rossi et al. (29)	Italy	2020	Retrospective single-center	109	–	–	CT	NSCLC	Radiomics (per-patient)	Radiology	A B
Chai et al. (30)	China	2021	Retrospective single-center	198 (54%)	402	58.1 (± 8.5)	CT	NSCLC	Radiomics (per-node)	Pathology	A B D
Wang et al. (31)	China	2019	Retrospective single-center	717	386	—	CT	NSCLC	Radiomics (per-node)	Radiology	B D

*A, Determine whether the patient has lung cancer; B, Determine whether the patient has non-small cell lung cancer; C, Determine whether the patient has malignant lung nodule; D, Determine whether the patient has lymph node metastasis*.

### Quality Assessment

**Figure 3:**
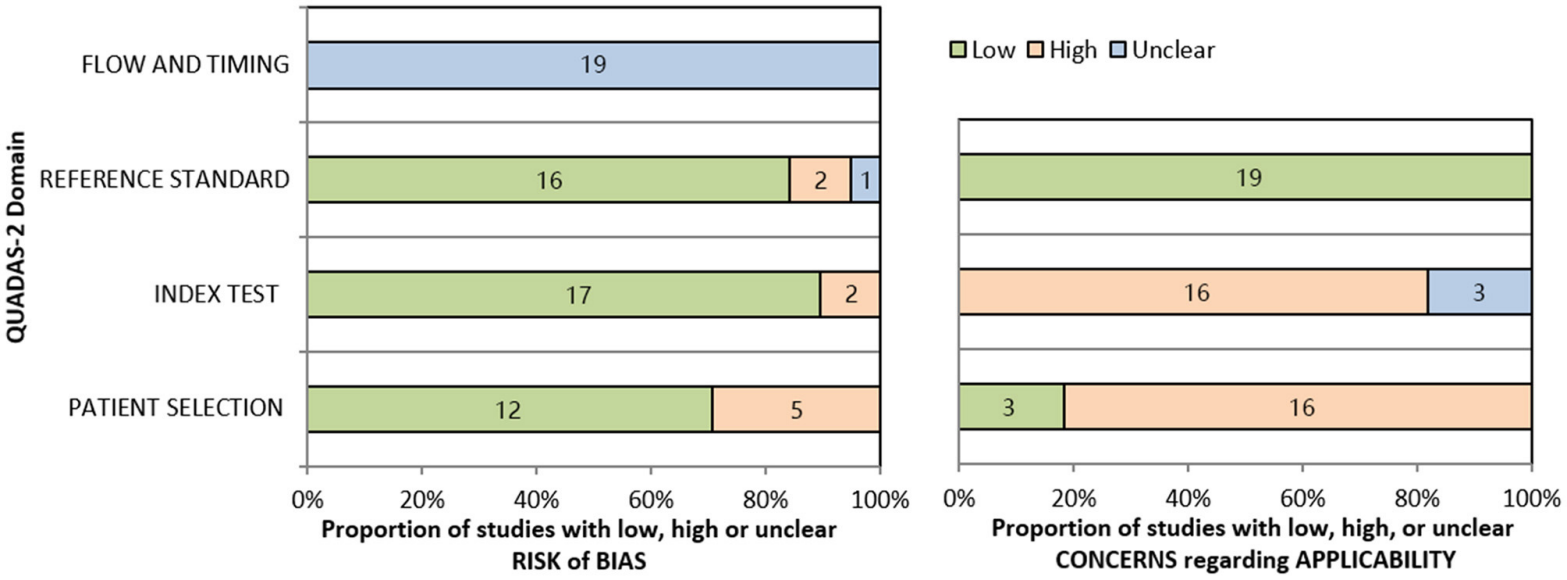
Summary of QUADAS-2 assessments of included studies.

**Figure 4:**
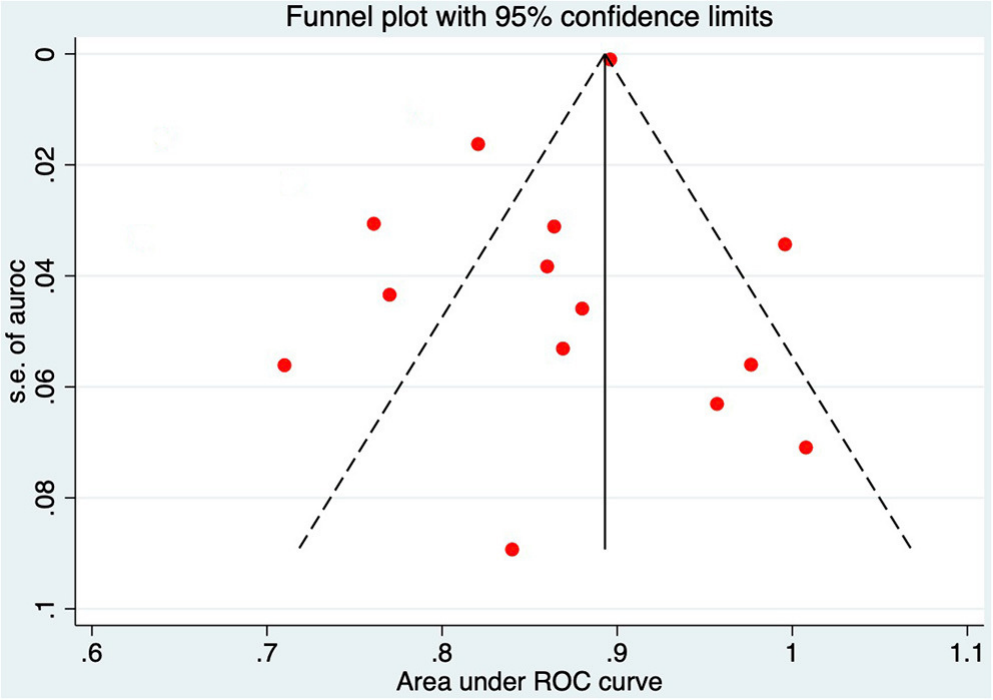
Funnel plot of the area under the receiver operating characteristic in 14 studies.

According to the QUADAS-2 tool, the summary of this study's assessment is shown in Figure 3. The risk of bias in patient selection was low in 12 (74%) studies and high in 5 (26%) studies. The risk of bias for the index test was high in 2 studies (10%) and low in 17 studies (90%). The risk of bias for the reference standard test was low in 16 studies (85%), high in 2 studies (10%), and unclear in 1 study (5%). Process and timing made the risk of bias unclear for all 19 studies. Table 4 shown individual evaluation of the risk of bias and applicability. Overall suitability issues are low. To assess the publication bias of the studies, a funnel plot was constructed (Figure 4). The shape of the funnel plot revealed asymmetry in the included studies, showing study heterogeneity.

**Table 4:** Quality assessment.

**Source**	**Risk of bias**								**Applicability concerns**		
	**Patient selection**			**Index test**			**Reference standard**	**Flow and timing**	**Patient selection**	**Index test**	**Reference standard**
	**Was the statistical management adequate?**	**Were the inclusion/exclusion criteria specified?**	**Was the type of study (retrospective or prospective) specified?**	**Were the imaging acquisition protocol and the segmentation method(s) detailed?**	**Was the image processing approach detailed?**	**Was the validation independent (i.e., no internal)?**	**Was the reference standard adequate?**	**Was there an appropriate interval between index test and reference standard?**			
Chetan et al. (1)	Yes	Yes	Yes	Yes	Yes	No	Yes	Unclear	Yes	Yes	Unclear
Parmar et al. (2)	Yes	Yes	Yes	Yes	Yes	No	Yes	Unclear	Yes	Yes	Yes
Sun et al. (3)	Yes	Yes	Yes	Yes	Yes	No	Unclear	Unclear	Yes	Yes	Yes
Ling et al. (4)	Yes	Yes	Yes	Yes	Yes	No	Yes	Unclear	Yes	Yes	Yes
Coudray et al. (5)	Yes	Yes	Yes	Yes	Yes	Yes	Yes	Unclear	Yes	Yes	Unclear
Xu et al. (6)	Yes	No	Yes	Yes	Yes	No	Unclear	Unclear	Yes	Yes	Yes
Baldwin et al. (7)	Yes	Yes	Yes	Yes	Yes	No	Yes	Unclear	Yes	Yes	Yes
Schroers et al. (8)	Yes	Yes	Yes	Yes	Yes	No	Yes	Unclear	Yes	Yes	Yes
Wang et al. (9)	Yes	No	Yes	Yes	No	No	Unclear	Unclear	Yes	Yes	Unclear
Leleu et al. (10)	Yes	Yes	Yes	Yes	Yes	Yes	Yes	Unclear	Yes	Yes	Yes
Ann et al. (11)	Yes	Yes	Yes	Yes	Yes	Yes	Unclear	Unclear	Yes	Yes	Unclear
Cong et al. (12)	Yes	Yes	Yes	Yes	Yes	Yes	No	Unclear	Yes	Yes	Yes
Botta et al. (13)	Yes	Yes	Yes	Yes	Yes	Yes	Yes	Unclear	Yes	Yes	Unclear
Botta et al. (13)	Yes	Yes	Yes	Yes	Yes	Yes	Yes	Unclear	Yes	Yes	Unclear
Wei et al. (14)	Yes	Yes	Yes	Yes	Yes	No	Yes	Unclear	Yes	Yes	Yes
Khorrami et al. (15)	Yes	Yes	Yes	Yes	Yes	No	Unclear	Unclear	Yes	Yes	Yes
Kirienko et al. (16)	Yes	Yes	Yes	Yes	Yes	No	Unclear	Unclear	Yes	Yes	Unclear
Rossi et al. (17)	Yes	Yes	Yes	Yes	Yes	No	Yes	Unclear	Yes	Yes	Unclear
Chai et al. (18)	Yes	Yes	Yes	Yes	Yes	Yes	Yes	Unclear	Yes	Yes	Yes
Wang et al. (19)	Yes	Yes	Yes	Yes	Yes	No	Unclear	Unclear	Yes	Yes	Unclear

### Diagnostic Accuracy

Of the 19 studies eligible for quantitative analysis, 14 used radiomics and 5 used deep learning. For each outcome, on a per-patient basis, pooled estimates including specificity, sensitivity, and AUROC were generated with 95% confidence intervals. The categorized data extraction for each study report is shown in Table 5. The type of lung cancer, malignant lung nodules, lymph node metastases, and deep learning or radiomics models discussed in each study were considered.

**Table 5:** Summary of AUROC for each study.

**References**	**Sensitivity, %**	**Specificity, %**	**Accuracy, %**	**AUROC**	**95%CI**	**Standard error**
Coroller et al. (13)	–	–	–	0.630	0.583–0.713	0.0331
Parmar et al. (14)	82.4	73.1	83.5	0.710	0.60–0.82	0.0561
Sun et al. (15)	–	–	–	0.770	0.69–0.86	0.0434
Ling et al. (16)	–	–	–	0.864	0.782–0.904	0.0311
Coudray et al. (17)	89.0	93.0	83.3	0.869	0.753–0.961	0.0531
Xu et al. (18)	–	–	63.5	0.670	–	–
Baldwin et al. (19)	99.57	28.03	40.01	0.896	0.876–0.915	0.0010
Schroers et al. (20)	86.95	93.25	88.89	–	–	–
Wang et al. (21)	64.04	58.97	61.47	0.640	0.61–0.67	0.0153
Leleu et al. (22)	–	–	72.6	–	–	–
Ann et al. (23)	79.9	75.2	65.8	0.761	0.59–0.71	0.0306
Cong et al. (24)	72.97	63.33	55.22	0.790	0.77–0.81	0.0102
Botta et al. (25)	–	–	–	0.840	0.63–0.98	0.0893
Wei et al. (26)	54.16	55.56	63.64	0.860	0.79–0.94	0.0383
Khorrami et al. (27)	61.34	57.16	63.81	0.880	0.79–0.97	0.0459
Kirienko et al. (28)	85.7	88.2	93.3	–	–	–
Rossi et al. (29)	100.0	66.7	85.7	0.850	–	–
Chai et al. (30)	–	–	95.3	–	–	–
Wang et al. (31)	–	–	72.4	0.712	0.678–0.770	0.0235

The data from radiomics models showed high heterogeneity, except for AUROC and the sensitivity of each node. After removing the literature with insufficient data, the pooled AUROC of the 7 studies determining whether a patient had lung cancer was 0.83 (95% CI 0.78–0.88; Figure 2A), and the pooled sensitivity and specificity were 0.838 and 0.653, respectively, indicating high heterogeneity (*I*^2^ = 65.3%, *p* = 0.008). For the 9 NSCLC studies that currently represent ~85% of lung cancer, the pooled AUROC of radiomics was 0.78 (95% CI 0.73–0.83; Figure 2B), and the pooled sensitivity and specificity were 0.782 and 0.715, respectively, with higher heterogeneity (*I*^2^ = 66.1%, *p* = 0.003). Among the six studies predicting benign or malignant pulmonary nodules, the pooled AUROC of radiomics was 0.79 (95% CI 0.77–0.82; Figure 2C), and the pooled sensitivity and specificity were 0.787 and 0.774, respectively, with heterogeneity relatively low (*I*^2^ = 9.7%, *p* = 0.354). Among the 6 studies that predicted the accuracy of LNM in lung cancer patients, the pooled AUROC of radiomics was 0.74 (95% CI 0.66–0.82; Figure 2D), and the pooled sensitivity and specificity were 0.661 and 0.598, respectively, with heterogeneity relatively high (*I*^2^ = 88.7%, *p* = 0.000).

## Discussion

During the diagnosis and treatment of lung cancer, many imaging data, such as CT, MRI, and PET, are generated. Doctors usually subjectively evaluate these data based on experience and make treatment plans (32). However, the features that doctors can observe from the image data with the naked eye are limited, and the potential of the image data is often not fully realized. Over the years, many researchers have tried to use complex mathematical and statistical algorithms to extract quantitative information that is hard to observe, even predicting cancer progression (33–35).

With the development of artificial intelligence technology, radiomics has emerged as the times require, using machine learning algorithms to mine high-throughput features from medical images and conduct modeling analysis. Increasing evidence shows that radiomics can be used for quantitative characterization of tumors for tasks such as disease diagnosis, treatment planning, and prognosis, which constitutes an important research direction for artificial intelligence technology in medical applications (36, 37). Radiomics is an emerging and rapidly developing field that integrates knowledge from radiology, oncology, and computer science and is an interdisciplinary subject that emphasizes the integration of medicine and engineering (38). With the rise of deep learning technology in recent years, the need for high precision and high stability in lung cancer staging has become more and more urgent (39).

To our knowledge, this is the first meta-analysis to summarize the diagnostic accuracy of deep learning and radiomics involving in lung cancer staging. We provided summarized data in this field and compared the identification effectiveness of lung cancer, tumor types, malignant lung nodules and lymph node metastase. In this article, the included studies mainly used radiomics (*n* = 14) rather than deep learning methods (*n* = 5). Of the five deep learning models, two were developed using transfer learning and three were developed using convolutional neural networks (CNN). Part of the reason there are relatively few deep learning models is that deep learning techniques are relatively new and prone to bias. The difference in the number of studies of the two AI models will lead to a significant deviation in the data ratio, affecting the ability comparison of the two models. Furthermore, most studies are retrospective in design, there are few prospective deep learning studies in lung cancer medical imaging staging, and most studies lack data and code availability. At the same time, most studies are single-center and use internal validation or resampling methods (cross-validation). However, internal validation tends to overestimate AUROC due to the lack of generality of the models, limiting the integration of AI models into clinical settings (40). Therefore, predictive models validated externally by using images from different hospitals are needed to create reliable estimates of the performance levels of other sites (41).

This systematic review performed a statistical assessment of pooled data collected from 19 studies. However, our findings must take into account some limitations. First, while comprehensive, our search may have missed some studies that could have been included. Second, we calculated estimates of diagnostic performance using limited data as several studies reported incomplete data. Third, there may be geographic bias because the included studies were from geographically different quantitative distributions. Finally, the type of scanner used for diagnosis, the imaging protocol, and the criteria for lung cancer staging may affect the accuracy of the results. In the future, the clinical benefit of diagnostic lung cancer staging models must be rigorously evaluated against current diagnostic criteria, as not all models are applicable in clinical practice (42, 43). Under the current hot spot of artificial intelligence development, more and more deep learning studies have shown that deep learning big data extracted from patients' medical images can have good clinical application value in tumor staging of patients. Therefore, we can combine deep learning features to establish a radiomics combined with deep learning diagnostic model, so that the accuracy of lung cancer staging diagnosis of patients can be improved.

## Conclusion

The models based on deep learning or radiomics have the potential to improve diagnostic accuracy in the pathological staging of lung cancer with the purpose of providing individualized preoperative non-invasive auxiliary prediction means for clinicians and realizing valuable prediction for patients to obtain better treatment strategy. Future studies are welcomed to use standardized radiomics features, more robust tools of feature selection and model development to further improve the diagnostic accuracy of artificial intelligence in lung cancer staging.

## Data Availability Statement

The original contributions presented in the study are included in the article/supplementary material, further inquiries can be directed to the corresponding author/s.

## Author Contributions

XZ and WH conceptualized the study. BH, YH, and MR collected the data. XZ, BH, and WH drafted the initial manuscript. ZC, ZZ, JM, LO, HC, and HG reviewed the included articles. YH and WH conducted the analyses. XZ, TL, and GL reviewed and revised the manuscript. All authors read and approved the final manuscript.

## Funding

This work was supported by the Chengdu Science and Technology Program, Grant no. 2021007 and Sichuan Science and technology plan, Grant no. 2018JY0356.

## Conflict of Interest

The authors declare that the research was conducted in the absence of any commercial or financial relationships that could be construed as a potential conflict of interest.

## Publisher's Note

All claims expressed in this article are solely those of the authors and do not necessarily represent those of their affiliated organizations, or those of the publisher, the editors and the reviewers. Any product that may be evaluated in this article, or claim that may be made by its manufacturer, is not guaranteed or endorsed by the publisher.

## Abbreviations

CT: Computer tomography
MRI: Magnetic resonance imaging
AI: Artificial intelligence
ML: machine learning
LNM: lymph node metastasis
QUADAS-2: Quality assessment of diagnostic accuracy studies tool 2
AUROC: Area under the receiver operating characteristic curve
NSCLC: non-small cell lung cancer.
